# The credibility struggle of mRNA vaccine rumors: A communication model to understand the impact of skepticism on public perception

**DOI:** 10.1177/22799036251407369

**Published:** 2025-12-23

**Authors:** Mia-Marie Hammarlin, Fredrik Miegel, Dimitrios Kokkinakis, Jullietta Stoencheva

**Affiliations:** 1 Department of Communication, Lund University, Sweden; 2 Department of Swedish, Multilingualism, and Language Technology, University of Gothenburg, Sweden; 3 School of Arts and Communication, Malmö University, Sweden

**Keywords:** COVID-19, mRNA vaccine, vaccine hesitancy, vaccine communication, vaccine rumor, Daniel Hallin, public health communication, public debate, social media, Twitter

## Abstract

**Objectives:**

This study explores the dynamics of vaccine rumors during the COVID-19 pandemic, particularly those surrounding messenger RNA vaccines. By employing and developing Hallin’s communication model of credibility spheres, we analyze how controversial ideas regarding vaccine safety gained public attention and challenged established vaccine narratives.

**Methods:**

The focal point of the investigation is the viral spread of a biomedical article from Lund University, which intensified existing vaccine rumors shared on Twitter, that, in turn, the authorities had tried to refute. Through a mixed methods analysis of Swedish-language tweets, reflecting a limited segment of the population’s opinions, we highlight persistent skepticism toward mRNA vaccines, characterized by fears of side effects, rushed development, and distrust in pharmaceutical companies.

**Results:**

The findings suggest that vaccine skeptics on Twitter leveraged the Lund medical article to legitimize their concerns, aiming to move their arguments from the Sphere of Deviant Vaccine Propositions into the Sphere of Legitimate Public Vaccine Debate, where they could be debated alongside mainstream views.

**Conclusion:**

We interpret the possible impact of the mRNA rumors shared on Twitter as an expression of an increasingly populistic society with a decreasing trust in democratic institutions and authorities, in which the constant flow of content via the internet reinforces the credibility of conspiracy theories.

## Introduction

In this article, we contribute to new ways of understanding vaccine rumors by placing current examples of rumors that circulated globally during the COVID-19 pandemic in a new theoretical framework. The aim of our investigation is to demonstrate how competing propositions in the form of rumors regarding COVID-19 vaccines claimed communicative credibility. We ask: What function do vaccine rumors have? What communication relationship can be detected between vaccine rumors and the public conception of vaccines as something inherently good? Employing and developing communication scholar Daniel Hallin’s model of credibility spheres,^1^ we will illuminate how more or less controversial conceptions regarding the safety of the vaccines claimed access to the legitimate, public debate. We term this model the Spheres of Vaccine Opinions’ Credibility.

We take as our case the fast spreading of a scientific article on Twitter, titled “Intracellular reverse transcription of Pfizer BioNTech COVID-19 mRNA vaccine BNT162b2 in vitro in human liver cell line,”^2^ written by a group of medical researchers, that spawned an already established vaccine rumor that mRNA vaccines alter the human genome. Our contribution is mainly theoretical, containing a secondary analysis of an already published mixed method study of Twitter conversations in Swedish from approx. 900 different users who published approx. 2000 tweets during a period of 10 months.^3^ Thus, here we give the reader a brief overview of the data while avoiding unnecessary repetition of details, providing space for the development of the rumor’s place in Hallin’s communication model.

### Significance for Public Health

To understand the dissemination of vaccine rumors is of significance for public health communication in at least three ways. First, our study shows that vaccine rumors can get new energy by state-of-the-art biomedical research, pointing to the fact that science can be used counter-epistemically by vaccine skeptics, problematizing the categorization of vaccine hesitant as a group that refutes science. Second, debunking rumors with more and accurate information might be a public health communication instinct, however, we show that research can be used to counter-debunking already officially debunked rumors – a rumor boomerang effect might occur. Thirdly, by introducing Hallin’s widely spread communication model into new areas of research, we visualize how vaccine rumors on social media might challenge the official vaccine debate, shedding light on today’s complex media landscape. We would like to think that the proposed communication model offers a tool for navigating this landscape and that it can be of use for both public health researchers and public health practitioners.

### Theoretical framework: Hallin’s model

Hallin’s^1^ model of three credibility spheres – Consensus, Legitimate Controversy, and Deviance – offers a useful lens for understanding how journalistic norms shape the boundaries of public debate. Originally developed to analyze war reporting, the model highlights how certain issues are framed as beyond dispute, others as open to debate, and still others as excluded from legitimate discourse^1^ (p. 117). It was constructed to better understand how ideals of objectivity within “neutral” American reporting during the Vietnam war (1957–1975) were influenced by other ideological notions, such as patriotism. Hallin argues that the journalists’ professional world can be interpreted as divided into these three regions, each of which is ruled by different journalistic standards^1^ (pp. 116–117). Highlighting boundary mechanisms for public debate, Hallin’s model is not only useful to understand journalistic work and ideals. Integrated in the vaccine rumor COVID-19 pandemic context that we study, it helps explore how holders of certain opinions that were kept outside of what we term the Sphere of Legitimate Public Vaccine Debate consequently used various tools to be judged more credible.^4^ To clarify how the spheres operate, we next unpack their meaning and relevance for our analysis.

In the middle, as a core, is the Sphere of Consensus. Hallin describes it as the region of “motherhood and apple pie”^1^ (p. 116); things that are good and thus not regarded by journalists or most of society as controversial and therefore in no need for debate. Here, the journalists are not inclined to find opposing views; rather they serve in the role of protectors of social and cultural consensus values. Bounding this sphere is the province of objectivity, termed the Sphere of Legitimate Controversy. It is where journalists are displaying their professionalism by handling legislative debates and electoral contests and similar matters that are recognized as worthy of public debate, striving to keep the balance between opposing views. Beyond these two spheres, outside of the Sphere of Legitimate Controversy, is the Sphere of Deviance, “[. . .] the realm of those political actors and views which journalists and the political mainstream of the society reject as unworthy of being heard.”^1^ (p. 117).

There is a fuzziness between the spheres, as Hallin^1^ (p. 117) points out, underscoring that social objects move between the three regions. However, it is reasonable to say that during the pandemic, COVID-19 vaccine critical voices were met by the majority as “those who violate or challenge the political consensus”^1^ (p. 117). Thus, they were either condemned or excluded from the public agenda, belonging to what we call the Sphere of Deviant Vaccine Propositions. From a public authorities’ communication perspective, the vaccine critical voices were mostly treated as misinformed, thus exposed as examples of wrong thinking. As earlier described, the idea that more and accurate information would change their disturbing views seems to have dominated their communication strategies.

**Figure 1.**
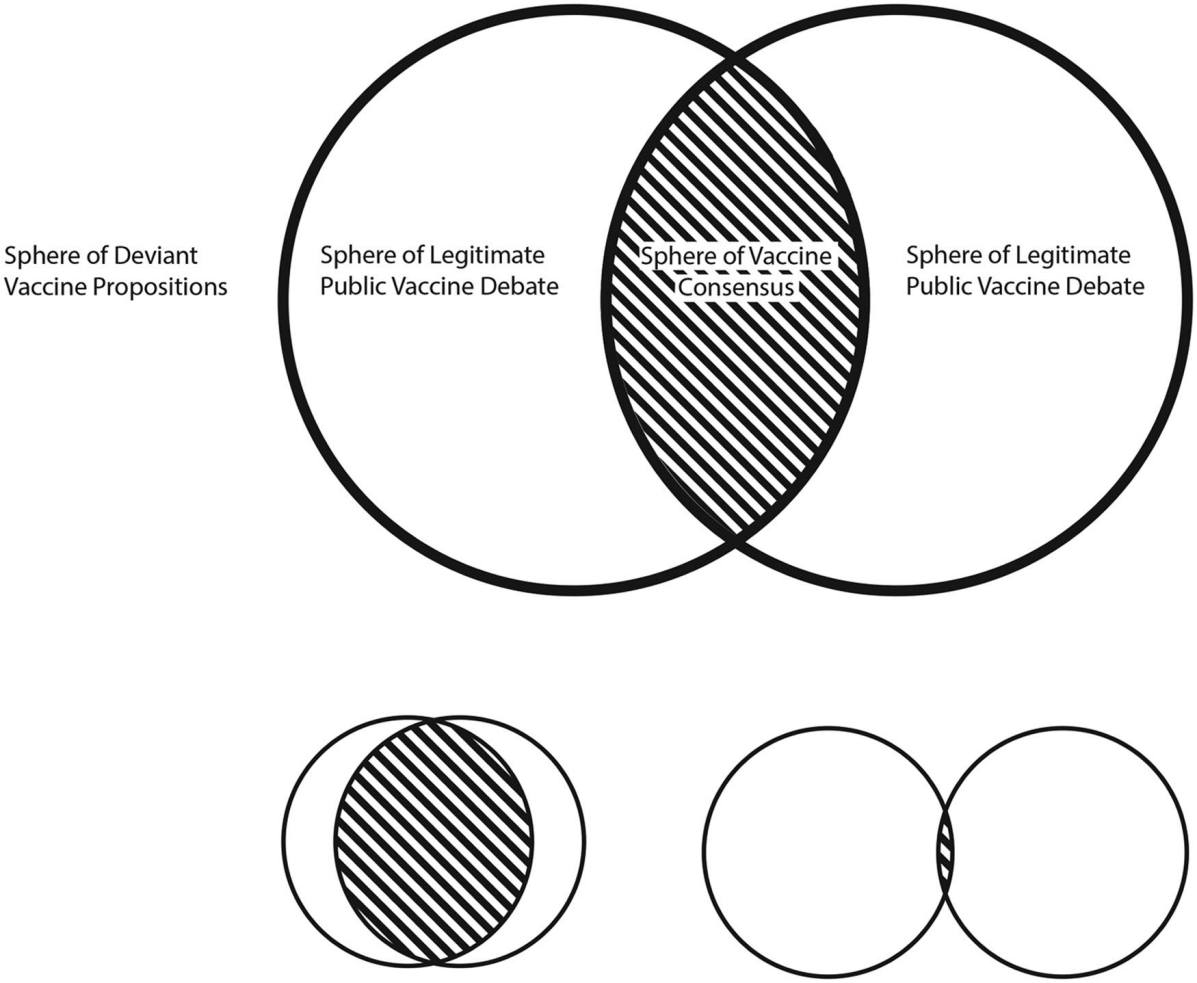
The Spheres of Vaccine Opinions’ Credibility demonstrate how the Sphere of Legitimate Public Vaccine Debate is not a status quo phenomenon, which also affects the Sphere of Vaccine Consensus. Depending on vaccine issue, the spheres vary in size. For example, in comparison with established vaccination programs, mass vaccination with new vaccines may increase deviant opinions concerning safety, affecting both the public debate and the vaccine consensus.

Ward,^4^ also building upon Hallin, extends this perspective by demonstrating how journalists engage in “boundary work” through source selection, framing, and format choices to maintain professional credibility. In his study of the 2009 flu vaccine controversy in France, Ward shows that most journalists aligned with dominant scientific actors, relegating radical vaccine criticism to the Sphere of Deviance while cautiously accommodating conditional critiques within Legitimate Controversy. This co-production of boundaries underscores that credibility is not static but negotiated through media practices – a dynamic that becomes even more salient in the digital era, where alternative platforms challenge traditional gatekeeping (Figure 1).

Integrating Ward’s insights with Hallin’s framework allows us to conceptualize mRNA vaccine rumor circulation as a struggle over these spheres, where actors seek to reposition their claims from deviance toward legitimacy.

### Messenger RNA vaccine rumors

During intense vaccination phases against COVID-19 between 2020 and 2021, numerous public health officials and authorities around the world were striving to debunk rumors related to mRNA vaccines. It usually sounded like this: “No, COVID-19 vaccines do not alter your DNA” (Australian Government’s Health Department)^5^ (https://www.health.gov.au/our-work/covid-19-vaccines/is-it-true#can-covid19-vaccines-alter-my-dna (accessed 03 June 2024)). In approximately the same wording UNICEF^6^ (https://www.unicef.org/eca/media/19996/file (accessed 03 June 2024)) stated that mRNA vaccines do not interfere with human DNA. Underneath the heading “Bust Myths and Learn the Facts about COVID-19 Vaccines,” the U.S. Federal Agency Centers for Disease Control and Prevention (CDC)^7^ wrote: “The genetic material delivered by mRNA vaccines never enters the nucleus of your cells, which is where your DNA is kept, so the vaccine does not alter your DNA.” (https://www.cdc.gov/coronavirus/2019-ncov/vaccines/facts.html (accessed 03 June 2024)).

It is well-known that fears regarding genetic mutations and immunity breakdown can be caused by the rapid bio-technological development in society^8^ (p. 33; see also Goldenberg^9^) that might hinder people’s willingness to, for example, take the BioNTech Pfizer and the Moderna jab. Thus, by providing more and accurate information the authorities identified people as “in need of injections of knowledge”^10^ (p. 257). Conceivably, this was not a very effective strategy for trying to convince skeptics or refusers who already distrust public health authorities,^9^ but still a somewhat reasonable communication action as rumors that vaccine would alter the DNA could have a negative effect on the willingness to take the shot^11^ (p. 456; Loomba et al.^12^). As Tworek et al.^13^ point out however, facts alone are insufficient for effective democratic health communication during crises. Communicating with compassion and emphasizing shared values while acknowledging individual agency are equally important for building trust and making health information feel reliable.

From our perspective, the authorities’ strategies to rebut mRNA vaccine rumors witness of the rumors themselves. The faster vaccine rumors are spread and circulated, for instance through social media, the greater the possibility that they will find their way into the more traditional domain of journalistic news, and thus also other established communication channels, such as public health agencies’ Public Relations offices. Public health communication trying to debunk rumors then inevitably becomes a part of folklore^14^ (p. 153). When this happens, the status of oral stories is raised from the rudimentary and popular to the authoritative and political, thus providing them with a certain meaning and value.^15^ With Rosanvallon, we also interpret the impact of the mRNA rumors as an expression of an increasingly populistic society with a decreasing trust in the democratic institutions and authorities, in which the constant flow of content via the internet reinforces the credibility of conspiracy theories and other alternative interpretations of reality^16^ (p. 42). Brazil^17^ and the U.S.^10^ (pp. 173–207) are examples of countries that were heavily exposed to politically distributed populist misinformation regarding COVID-19 vaccines with detrimental effects on public health.

This points toward the democratic challenges of the current development of media and communication in society, which encourages us to investigate the civic aspects of vaccine rumors meaning that we regard them as a form of *civic talk*^18^; speedy, alarmistic, political conversations where citizens discuss real matters and problems that are important to them. With studies that investigate messenger RNA vaccine rumors as expressions of distrust,^11,19^ rather than offering communication strategies to debunk them,^20,21^ we strive to understand vaccine rumors by suggesting that they are means of quickly seeking answers where official sources are deemed doubtful, bio technical achievements are questioned, and the public vaccination consensus is challenged in turbulent, pandemic times. This does not mean that the public spreading mRNA rumors are rejecting the cultural authority of science^19^ (p. 923). Instead, we show how state-of-the-art vaccine research can be used by citizens to challenge the official communication strategy to refute vaccine rumors.

While our aim is not to quantify behavioral patterns, prior research shows that rumors can undermine vaccination programs and public trust, as seen in cases like Denmark’s human papillomavirus (HPV) vaccine debate, where skepticism and rumors about severe side effects led to a sharp decline in coverage^22^ (see also Larson^23^ for more examples). Such instances illustrate why understanding the communicative dynamics of rumors is crucial: they can destabilize consensus and affect health behaviors.

### When research becomes rumors

Having presented Hallin’s model, we will now take a look at what happened with the biomedical article “Intracellular reverse transcription of Pfizer BioNTech COVID-19 mRNA vaccine BNT162b2 in vitro in human liver cell line.”^2^ The title of this scientific paper, written by a group of researchers at Lund University, Sweden, published in February 2022, is somewhat a riddle for people with scarce knowledge in biomedicine, but the expert knowledge needed to interpret the results of the study did not hinder it from going viral. At this specific time, Sweden saw its sharpest peek of COVID-19 cases since the outbreak with more than 250,000 people being infected by the disease (Figures from the Swedish Public Health Agency: https://www.folkhalsomyndigheten.se/faktablad/fall-covid-19/ (accessed 27 August 2024) Sweden has approx. 10.5 million inhabitants). Six months after the publication, the article had been viewed in full text more than 1.1 million times. By spring 2024, it had been viewed 2.1 million times, making it one of the most popular research outputs ever tracked by the journal’s attention metric tool. It was particularly shared on Twitter (In July 2023, the platform changed its name to X, but as our investigation encompasses material from before this change, we will refer to Twitter instead of X and tweets instead of x’s.), where 97% of the 89,500 tweets – authored by 57,000 users with a combined 14 million followers – were written and disseminated by members of the public. Notably, this wave of interest did not have a counterpart in the traditional media where the study received minimal attention, though it did garner some coverage in alternative media outlets.

What was this mRNA vaccine study about? The question that Aldén and colleagues wanted to answer was: “Does the Pfizer-BioNTech mRNA vaccine get converted to DNA or not?” When answering the question affirmatively – the vaccine did get converted to DNA in the experiment – the research team, foreseeing attention by the public, quickly contacted Lund University’s press center to arrange a Q&A.^24^ One of the authors, Yang de Marinis, Associate Professor with an expertise in Epigenetics, said:

This study does not investigate whether the Pfizer vaccine alters our genome. Our publication is the first in vitro study on the conversion of mRNA vaccine into DNA, inside cells of human origin. We show that the vaccine enters liver cells as early as 6 hours after the vaccine has been administered. We saw that there was DNA converted from the vaccine’s mRNA in the host cells we studied.(Lund University’s website: https://www.lunduniversity.lu.se/article/qa-covid-19-vaccine-study-gains-attention (accessed 20 August 2024))

More studies need to be done, particularly on living human bodies, the researchers concluded. Professor Magnus Rasmussen clarified this in the following way: “These findings were observed in petri dishes under experimental conditions, but we do not yet know if the converted DNA is integrated into the cells’ DNA in the genome – and if so, if it has any consequences.” However, the communication precautions taken did not hinder the study from giving new energy to the – in vaccine skeptic groups – widespread idea that mRNA vaccines do alter the human genome. This is not the first time that a medical study has been misrepresented by part of the public to warn about mRNA vaccines presumed effects on the DNA. An article^25^ written by a group of scientists, among them researchers from the Massachusetts Institute of Technology (MIT), that was published during the COVID-19 pandemic attracted similar attention, causing debates both within and outside of academia (Information about the study and the subsequent debate can be found in Hackethal.^26^). Lund University and MIT, respectively, have very high academic credibility. Besides this, factors that may have influenced the fast spreading of the studies on social media are (a) sensationalized messages by posters in times of uncertainty; (b) that evoke fear and other negative emotions; (c) which increase people’s willingness to engage themselves in interpersonal communication.^27^

## Design and methods

We have designed the analysis in the following way: first, we describe the Twitter material that we have studied and the methods that we use; three, zooming out again, we employ Hallin’s credibility model to demonstrate how mRNA rumors, supported by scientific arguments, can be used by vaccine skeptics when trying to enter the official vaccine debate.

## Mixed methods

To better understand the course of these events, and to be able to answer our research questions, our team studied Swedish-language tweets discussing mRNA vaccines.^3^

We focused on Twitter due to it being the platform where Aldén et al.’s^2^ publication gained the widest traction, in Sweden and elsewhere. Compared to other social media channels, such as Facebook, Twitter hosted a significantly higher volume of posts referencing the study. Twitter’s design as a real-time, text-based network also attracts political actors, journalists, and opinion leaders, making it a key arena for shaping and amplifying public narratives.^28^ This combination of volume and influence positioned Twitter as the most relevant platform for examining the vaccine rumor’s communicative dynamics.

Sweden represents a particularly relevant case in this context for two reasons: (1) Sweden had one of the highest COVID-19 vaccination rates in Europe and globally (approx. 85% had received 1 dose in November 2021, cf. Rönnerstrand^29,30^), rendering skepticism particularly noteworthy in this context. Thus, (2) by analyzing the spread of a biomedical article, stemming from a Swedish university, we provide insights into how vaccine rumors interact with a high-trust welfare society and how scientific research can be reframed in such a setting.

The data contained 2028 unique mRNA-tweets in Swedish from 903 different users, published between February 10, 2022 to November 10, 2022, encompassing the most intense phase of the popular dissemination of Aldén et al.’s^2^ study. The inclusion criteria required that tweets were written in Swedish, explicitly referenced mRNA vaccines, and were original posts within the designated study period. Exclusion criteria included retweets without added commentary, tweets containing fewer than three words, duplicates, and posts unrelated to vaccine discourse despite containing the search terms. We then followed a mixed methods sequential explanatory research design in a two-step process^31^; an initial computational distant reading analysis followed by a close qualitative reading (For a detailed explanation of Material and methods, please see Hammarlin et al.^3^). This research design offers several advantages by providing a more comprehensive and holistic understanding of the users’ views and thoughts on mRNA vaccines, enabling us to explore both the breadth and the depth of the discussions.^31^ The reporting of this mixed methods study conforms to the Equator guideline GRAMMS^32^ (see the Appendix).

By searching the keywords m-RNA or mRNA, the tweets were collected. Each tweet was then preprocessed in various ways. During a so-called normalization process we identified token variants such as “mrna vax,” “mrna vaccin,” and “mrnavaccin,” which were converted to a single uniform format, here “mrna-vaccin.” Furthermore, the dataset was tokenized by separating punctuation and metadata. Multiword expressions were recognized, and their contiguous components were joined with an underscore prior to further processing. Stopwords, like *and*, *on*, *is*, *to* (English translations of Swedish stopwords) were removed as they tend to be over-represented. Finally, posts with less than three words were removed, and the rest was used as input to the topic modeling software.

No formal ethics approval was required because the study relies exclusively on publicly available online data. Following the Association of Internet Researchers^33^ Ethical Guidelines, we protect the Twitter users’ identity by not mentioning their names or alias. On plus, all quotes are provided as translations from the original Swedish into English, which make them difficult to retrieve.^34^

As the focus was on topic modeling and qualitative interpretation of vaccine-related discourse, no formal statistical analysis was conducted in this study. Topic modeling approaches, such as Latent Dirichlet Allocation (LDA), are primarily exploratory and do not inherently rely on inferential statistical testing. Instead, they aim to identify latent semantic structures across a corpus. While studies using Twitter data often complement topic modeling with descriptive statistics (e.g. frequency distributions, or temporal trends) and sometimes with inferential tests to compare groups or time periods, our analysis remained limited to computational modeling and qualitative interpretation. This choice aligns with our objective of capturing the breadth and depth of the discussions rather than testing statistical hypotheses.

### Topic modeling

Topic modeling is an unsupervised classification of textual documents which allows researchers to get a bird’s-eye view of large-sized text data. Furthermore, we applied an extension to latent Dirichlet allocation (LDA),^35^ called structural topic model (STM; For the structural topic modeling we used the R package stm, version 1.3.6), that admit the integration of covariates, such as date and time of publication. Thereby, STM can gain valuable insights and understanding on how topics evolve. Several diagnostic aspects of the topic modeling were evaluated to decide the number of topics, *k*, to use. We ran multiple models with a varying number of *k* values, ranging from 2 to 40. We evaluated them using standard diagnostic criteria, including semantic coherence, held-out likelihood, and exclusivity, which balance interpretability and fit: coherence ensures that top words co-occur, while exclusivity ensures topics remain distinct. Based on these diagnostics, we selected nine topics, which offered the best trade-off and produced thematically meaningful clusters. Manual inspection of topic-word distributions and tweet assignments further confirmed that the chosen topics captured the dataset’s dominant themes.

**Figure 2.**
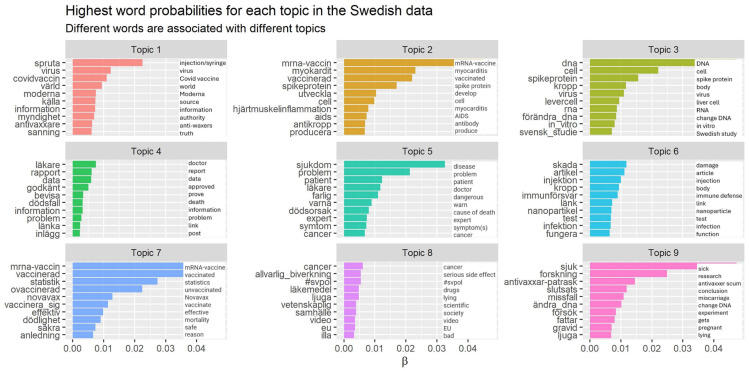
The top-10 probable words for each of the nine generated topics in the text corpus.

After these processes, we took a closer look at the results of the STM in which general themes emerged based on the topics identified. Structural topic models are quantitative by nature and deal with the challenge of retrieving thematically similar documents – in our case tweets – from the data. To achieve this objective, the document input to topic modeling is de-contextualized with the aim of developing themes. Figure 2 shows the top-10 probable words for each of the nine generated topics.

We could see that people clearly highlighted their concerns on the safety of the mRNA technology. Some common subjects that emerged include doubts about perceptions of rushed vaccine development; mistrust in “big pharma”; and fear of vaccine side effects, highlighting the long-term effects of potential adverse reactions. Concerns about personal autonomy and freedom were also prominent. Notably, the World Health Organization (WHO) was mentioned in a throughout negative tone of voice, sometimes paired with antisemitic imaginaries and conspiracy theories.

#### A qualitative refinement of the quantitative selection

Following on the quantitative analysis, we used the data analysis software NVivo to complement the topic modeling with additional qualitative thematic analysis. The qualitative component of this mixed-methods design offered important advantages by enabling a deeper understanding of the nuances of how credibility was rhetorically negotiated in tweets than what topic modeling alone could reveal. As Gillies et al.^36^ argue, topic modeling can act as a “seed” for qualitative inquiry by mapping broad semantic patterns that help researchers navigate and familiarize themselves with large-scale datasets. In our case, the topics derived from the structural topic model provided an initial map of Twitter’s conversation landscape surrounding mRNA vaccines in Sweden at the time, while the qualitative analysis grounded the analytical interpretation in the social and communicative logics of the tweets themselves. The NVivo software facilitated the collaborative organization and coding of the tweets into analytical categories, their iterations and refinements. It also enabled real-time coding comparisons, which allowed us to resolve ambiguities and strengthen internal consistency through regular intercoder discussions within the group.

First, we conclude that positive vaccine mRNA comments were few and far between; an interesting finding in itself considering the platform’s inherent opportunities to engage in debates and the significantly different societal consensus on the issue of vaccination; in comparison with many other countries, Sweden is a strong welfare state with a documented high confidence in vaccines, COVID-19 vaccines being no exception. This apparent dominance of negative voices may partly reflect a spiral of silence effect, where users who hold pro-vaccine views refrain from posting due to perceived minority status or fear of confrontation in polarized threads.^37^ In other words, the absence of positive comments does not necessarily indicate their absence in society but rather a reluctance to engage publicly when the discourse appears overwhelmingly critical. The most common, interrelated themes in the tweets were, in descending order:

- lack of confidence in vaccine safety;
- fear of long-term side effects;
- perceptions of rushed development processes;
- threat to personal autonomy and freedom;
- distrust in pharmaceutical companies, such as Pfizer;
- distrust of wealthy individuals such as Bill Gates and George Soros, who by their power aim as their goal of the global and total control of humanity.

This predominantly negative attitude towards COVID-19 vaccinations expressed on Twitter in Swedish in 2022 has been confirmed and explored by other studies (Beirakdar et al. 2023)^36^. Several of these topics expressed in Swedish on Twitter are also common in other cultural contexts,^38^ signaling that criticism against vaccines is largely spread and formed across national borders through, among other means, social media. It is worth noting that, with exception of the last and (to a certain extent) first theme, these themes capture reasonable concerns regarding vaccine safety. It is precisely this legitimacy that gives fuel to the misinformation spreading during the COVID-19 pandemic and vaccination periods, as the fuzziness between spheres enables reframing disinformation into legitimate critique.

Second, to a high degree the tweeters were building their argumentation on their own interpretation of the results from the medical article from Lund University,^2^ often mixing this and other scientific sources with unsupported claims to display their distrust, such as vaccines being used for population control (“Genetic substances are given to control people”), or that vaccines contain poisonous ingredients (“The mRNA vaccines contain metals that form inorganic ‘plants’ in the bloodstream”). These topics seem at least to some degree to be part of globally spread populist, antagonistic disinformation and hate speech typical for white nationalists’ communication online, not least on Twitter/X under CEO Elon Musk’s “free speech absolutist” strategy^39^ (see also Bennett and Livingston^40^), but it would probably be a mistake to politically label all mRNA vaccine skeptics in this way. Their views can also be read as expressions of popular fears and anxieties.^11^

In the following section, we will further explore the content of the tweets through our developed version of Hallin’s model. It is worth noting that our proposed model is not intended as a measurement instrument but as a conceptual framework for exploring credibility dynamics in vaccine discourse. Similar to Hallin’s original spheres of credibility, it functions as an interpretative tool rather than a statistical model. Its purpose is to illuminate rhetorical and communicative strategies – how actors seek legitimacy and reposition their views – rather than to quantify movement between spheres. In this sense, the model offers a lens for analyzing discursive struggles, enabling researchers and practitioners to reflect on the shifting boundaries of consensus, controversy, and deviance in public health communication. The model’s generalizability lies in its conceptual and theoretical applicability for analyzing comparable occurrences elsewhere, rather than in its empirical results as such which, as most social scientific research, is context dependent.

### The spheres of vaccine opinions’ credibility

During a severe societal crisis marked by fear and uncertainty, such as the COVID-19 pandemic, the Sphere of Consensus tends to become an increasingly impenetrable core of beliefs, strengthening the sense of a collective “we.”^41^ It is however the credibility of such authorities that is being questioned in the Twitter threads. By suggesting the unreliability of an acknowledged authority such as WHO (which strived to refute the mRNA rumor as earlier described), this kind of questioning functions to weaken the general vaccine consensus, making it less stable, signaling the need for opposing opinions about WHO to enter the Sphere of Legitimate Public Vaccine Debate. Only when having become a part of this sphere will people’s concerns be taken seriously and listened to by the majority, which in the end would bring us further to the truth, or at least this is what the skeptics believe. During the pandemic, this sphere included, for example, different opinions regarding how to weigh the risks of known side effects (such as myocarditis) against the benefits of preventing the spread of the infection, cost-benefit calculations concerning the utility of vaccination programs, and multiple other topics where no unequivocal answers or stances among experts and authorities were available. Arguably, the function of the mRNA vaccine rumor enhanced by Aldén et al.’s^2^ article was to *widen* the Sphere of Legitimate Public Vaccine Debate to encompass not only criticism against WHO but the fear and worry that mRNA vaccines lack reliability and might be dangerous to the health, as can be seen in the following examples:

Some covid vaccines contain a new technology called mrna, gene modifier, this is the first time this technology has been tested on a large scale. A study from Lund University shows that these gene modifiers have in some cases ended up in the wrong places in the body after injection.

[link to study] According to a study from Lund University, the spike protein in Moderna’s and Pfizer’s covid19 vaccine is converted in the liver with genetic changes as a result. Complicated connections for a layperson, but the process seems capable of causing autoimmune diseases.

Symbolically speaking, the tweeters were pushing the borders of the spheres, wishing for mRNA vaccines’ (imagined) side effects to be taken seriously and debated on equal terms by experts in established newsrooms. When this is hindered by different stakeholders, power relations are questioned, as in this typical tweet: “Media and Government REFUSE TO SHARE THIS STUDY FROM LUND” (capital letters in original). “System media” is a term frequently used among Swedish vaccine skeptics to highlight power dynamics within media and journalism, underscoring their assumed reliance on authoritative power, such as the government, and their perceived unwillingness to challenge consensus regarding COVID-19 vaccines and other political issues. The authorities’ ambition however was to communicate the view of the mRNA vaccines as safe, belonging to the Sphere of Vaccine Consensus, with the consequence that mRNA rumors proposing that the vaccines were hurriedly produced and insufficiently tested were kept in the Sphere of Deviant Vaccine Propositions.

One finding that we find particularly important is that both pro- and anti-vaccinationists can be said to place science in the Sphere of Consensus by acknowledging its societal value as something inherently good. In fact, the skeptics appear to imagine science as being undermined by conflicts of interest and coverups by the media and the authorities, and thus, regard themselves as defenders of science integrity. We also observe that most of the tweets leave little room for doubt. Instead, they were conveyed with confidence in their validity. One Twitter user writes:

A shocking new study conducted at Lund University in Sweden has *confirmed* that mRNA nanoparticles from Pfizer’s Covid-19 vaccine enter human cells and are reverse-transcribed into DNA, causing a permanent change to the person’s genetic code. [our italics]

Another states:

[…] it’s gene therapy that has been *proven* to change the recipient’s DNA. A Swedish study from Lund published in Current issues of Molecular Biology *shows* this. [our italics]

We also note that the Aldén et al. study was mentioned in mRNA related discussions even in cases where the topic at hand was not directly related to the study’s findings.

Seen from the other side of the coin, even when referring to scientific studies, the tweeters’ arguments are judged by the majority as lacking credibility, thus belonging to the realm of contentions and allegations with no support in credible institutions and authorities required to be taken seriously in the public political discourse. The arguments in this sphere – the Sphere of Deviant Vaccine Propositions – tend to be, as described earlier, diminished, ridiculed, or simply ignored in the public debate, and those who assert them discharged as conspiracy theorists, science deniers or fact resistant tin foil hats. It is unsurprising that being dismissed from public debate causes negative reactions and emotions among the vaccine hesitant, such as increased fear, frustration, and anxiety, as well as a need to “proof” that they are right, and to find other communication platforms.

In this particular case the borders between the spheres seem to have maintained more or less intact; as already mentioned, the wave of interest on Twitter did not have a counterpart in the traditional media where the Aldén et al.^2^ study received very little attention. Would it however have been downright negative if deviant vaccine propositions managed to enter the Sphere of Legitimate Public Vaccine Debate? A notable recent case illuminating this issue involves the Gardasil vaccine against HPV included in the Danish childhood vaccination program in 2009. Initially successful, the program saw a rapid and significant decline in coverage 5 years later following Danish media reports featuring stories from girls who believed they experienced severe side effects from the vaccine.^22^ A prominent television documentary further fueled the rumors and skepticism these side effects reports generated. The documentary was widely discussed in traditional media and shared through social media, leading to more testimonies from girls suspecting serious adverse effects from the vaccine. By analyzing national newspaper content focusing the Danish HPV debate 2008–2018, Agergaard et al.^42^ (p. 12) conclude that the “[. . .] media coverage of adverse reactions and risks associated with HPV vaccination was in fact diverse”; it included reporting of suspected side effects by the “HPV girls” but also experts disagreeing with and challenging the girls’ narratives.

From our interpretation of Hallin’s spheres of credibility, on the one hand, it is possible that Denmark faced these challenges because of vaccine criticism becoming a legitimate topic of discussion in traditional media. The balanced reporting, rather than being beneficial, may have contributed to decreased trust in the HPV-vaccine. As explained by Hopp and Ferrucci^43^ (p. 504), “Once awarded, legitimacy is difficult to revoke.” On the other hand, it is plausible that too much conformity and consensus in vaccine communication can lead to suspicion among some (cf. Harambam^44^).

## Discussion

We comprehend the function of the mRNA vaccine rumor, reinforced by Aldén et al.’s study, as a destabilizer of the Spheres of Vaccine Opinions’ Credibility, facilitated by media and communication technology. Thus, the fast circulation of the medical article became a lever for vaccine skeptical citizens to push against the borders of the Sphere of Legitimate Public Vaccine Debate, stretching its limits and redefining its contours.

However, to question authorities and power holders is a crucial part of how citizens in a democratic society are civically engaged and hence important for vivid, civic talk. Routinely dismissing the concerns and apprehensions going on in the outskirts of the public vaccine discourse would be a mistake. But, in our interpretation of Hallin’s model it is in the Sphere of Legitimate Controversy that the civic culture of a society is located. If inaccurate vaccine rumors manage to enter and gain foothold in this sphere, they at the same time become part of the public discourse taking place in established news channels about vaccine and vaccination. The consequences of such a development are unfortunate and potentially harmful for the civic culture of a society, as have been pointed out by several contemporary thinkers.^16,45^ Paradoxically however, the experience of a too coherent pandemic narrative being formed by public officials and politicians may also be fertile ground for doubt.

We conclude that the uninterrupted flow of content on the internet has made the spheres of credibility less solid. It has become easier for ideas from the Sphere of Deviance to make inroads in the Sphere of Legitimate Controversy from which challenges against the Sphere of Consensus can be made. According to some, the Sphere of Deviance is in fact shrinking in the current, digital times, regardless of debate topic.^43^ The case of a well-known mRNA rumor shared on Twitter, that was strengthened by a medical article, is an example of how social media users’ deviant vaccine opinions might end up challenging the vaccination consensus.

This study has limitations: the empirical material encompasses Swedish language content only, making it difficult to draw conclusions regarding the meaning in the total amount of tweets, written in English and several other languages. Still, the dataset remains valuable if one considers voices from the Nordic countries as integral to the global – indeed transnational – exchange of opinions, which Twitter clearly facilitates.

In addition, no formal sample size calculation was conducted, which should be noted as a limitation, although the dataset was still considered sufficiently large to capture relevant patterns.

As we have shown however, medical researchers need to be prepared for their findings to be misinterpreted under certain circumstances. This should not impact their research, but it may necessitate new communication strategies and increased internal support within workplaces. This is particularly important when a study goes viral, potentially leaving researchers with an uncanny feeling as though they have lost control over their results.

Additionally, public health officials need to consider this type of civic engagement in their communication strategies, where vaccine skeptics are understood as engaged citizens who, in available channels, discuss problems that matter to them. In our view, identifying the public as in constant need of injections of medical knowledge will not take us all the way. Rather than relying on the traditional “information deficit” approach, future communication strategies should prioritize dialogic and trust-oriented practices. This includes integrating empathetic messaging, acknowledging complexities, and avoiding overly uniform narratives that risk fostering suspicion. Additionally, cooperation between public health agencies and academic institutions, fostering interdisciplinarity, is essential to prepare for viral dissemination of researchers’ work. We would like to think that our model of the Spheres of Vaccine Opinions’ Credibility can be used as a tool in the process of developing new public health communication methods and collaborations.

## Results

The study offers a communication model that functions as an analytical tool, highlighting different vaccine opinions’ credibility. Analysis of over 2000 Swedish-language tweets revealed dominant skepticism toward mRNA vaccines following the viral dissemination of Aldén et al.’s^2^ biomedical study. Topic modeling identified nine thematic clusters, with the most prevalent concerns including vaccine safety, fear of long-term side effects, perceptions of rushed development, and distrust in pharmaceutical companies and global elite persons. Qualitative refinement confirmed that positive sentiments toward mRNA vaccines were rare, and that Twitter users interpreted the biomedical mRNA-study as evidence supporting their skepticism. Tweets often blended scientific references with unsupported claims, such as genome alteration and population control, reflecting a fusion of legitimate concern and misinformation. Overall, these narratives were framed so that they potentially could challenge the general vaccine consensus in the Swedish society. Despite the widespread attention on Twitter, the discussion received minimal coverage in traditional media, suggesting limited permeability between digital rumor circulation on Twitter and mainstream journalistic discourse in this particular case.

## Conclusion

This study shows how vaccine rumors, fueled by scientific state-of-the-art-research, can disrupt public trust and challenge the boundaries of credible vaccine debate. Developing Hallin’s^1^ classic communication model, we demonstrate how skeptics seek legitimacy by reframing their views as worthy of public debate. As digital platforms blur the lines between vaccine consensus and controversy, public health communication must adapt, acknowledging public concerns while safeguarding scientific integrity. Our model offers a tool for navigating this evolving media landscape.

## Appendix

**Appendix.** GRAMMS checklist for piloting the credibility struggle of mRNA vaccine rumors: a communication model to understand the impact of skepticism on public perception.

Item	Assessment
1. Describe the justification for using a mixed methods approach to the research question.	The study uses mixed methods to explore both the breadth and depth of public skepticism toward mRNA vaccines on Twitter. This approach allows for a nuanced understanding of how scientific research can fuel vaccine rumors and challenge public health narratives.
2. Describe the design in terms of the purpose, priority, and sequence of methods.	The purpose was to investigate how a biomedical study^2^ influenced vaccine-related discourse. A sequential explanatory design was used, with quantitative topic modeling conducted first to identify themes, followed by qualitative analysis to interpret and refine these themes. Priority was given to the qualitative interpretation to understand the civic and communicative dimensions of the rumors.
3. Describe each method in terms of sampling, data collection and analysis.	Quantitative:
	- Sampling: 2028 Swedish-language tweets from 903 users, selected based on relevance to mRNA vaccines and the study period.
	- Data Collection: Tweets were collected using keyword searches and preprocessed (normalization, tokenization, stopword removal).
	- Analysis: Structural Topic Modeling (STM) using R package stm, with covariates like publication date.
	Qualitative:
	- Sampling: Same dataset as above.
	- Data Collection: Tweets were manually coded using NVivo.
	- Analysis: Thematic categorization and interpretation, with intercoder reliability ensured through team discussions.
4. Describe where integration has occurred, how it has occurred and who has participated in it.	Integration occurred during interpretation. Themes identified through STM were refined and contextualized through qualitative coding. The combination revealed how scientific references were used to legitimize skepticism and how rumors had the potential to move between what we term The Spheres of Vaccine Opinions’ Credibility.
5. Describe any limitation of one method associated with the presence of the other method.	The study is limited to Swedish-language tweets, which restricts generalizability across linguistic and cultural contexts. No formal sample size calculation was conducted. Additionally, topic modeling is exploratory and does not include inferential statistical testing.
6. Describe any insights gained from mixing or integrating methods.	Mixing methods revealed that while quantitative modeling identified dominant themes (e.g. safety concerns, distrust in pharma), qualitative analysis showed how these themes were rhetorically framed to challenge vaccine consensus. The integration highlighted the civic nature of rumor-spreading and the strategic use of scientific discourse to gain credibility.
